# Cannabinoids on the Brain

**DOI:** 10.1100/tsw.2002.139

**Published:** 2002-03-09

**Authors:** Andrew J. Irving, Mark G. Rae, Angela A. Coutts

**Affiliations:** Neurosciences Institute, Department of Pharmacology and Neuroscience, University of Dundee, Scotland, DD1 9SY, UK; 2Department of Biomedical Sciences, University of Aberdeen, Scotland, AB25 2ZD, UK

**Keywords:** cannabis, cannabinoids, marijuana, CB_1_, endocannabinoids, central nervous system (CNS), brain, synaptic transmission, synaptic plasticity, function, reward, cognitive function, attention, pain, neurodegeneration, epilepsy, appetite, therapeutic potential, review

## Abstract

Cannabis has a long history of consumption both for recreational and medicinal uses. Recently there have been significant advances in our understanding of how cannabis and related compounds (cannabinoids) affect the brain and this review addresses the current state of knowledge of these effects. Cannabinoids act primarily via two types of receptor, CB_1_ and CB_2_, with CB_1_ receptors mediating most of the central actions of cannabinoids. The presence of a new type of brain cannabinoid receptor is also indicated. Important advances have been made in our understanding of cannabinoid receptor signaling pathways, their modulation of synaptic transmission and plasticity, the cellular targets of cannabinoids in different central nervous system (CNS) regions and, in particular, the role of the endogenous brain cannabinoid (endocannabinoid) system. Cannabinoids have widespread actions in the brain: in the hippocampus they influence learning and memory; in the basal ganglia they modulate locomotor activity and reward pathways; in the hypothalamus they have a role in the control of appetite. Cannabinoids may also be protective against neurodegeneration and brain damage and exhibit anticonvulsant activity. Some of the analgesic effects of cannabinoids also appear to involve sites within the brain. These advances in our understanding of the actions of cannabinoids and the brain endocannabinoid system have led to important new insights into neuronal function which are likely to result in the development of new therapeutic strategies for the treatment of a number of key CNS disorders.

## INTRODUCTION

The hemp plant *Cannabis sativa,* often used in the herbal form (marijuana) or as resin (hashish), has a long history of consumption, both for recreational and medicinal uses; indeed, cannabis has widespread actions in the body, including the modulation of a number of key processes within the central nervous system (CNS). Some of these actions are described as undesirable or unpleasant whereas others have considerable therapeutic properties. Recently there have been significant advances in our understanding of how cannabis and related compounds (cannabinoids) affect the CNS.

The term *cannabinoid* originally described a family of chemically related 21-carbon alkaloids found uniquely in the cannabis plant, of which $\Delta^9$-tetrahydrocannabinol ($\Delta^9$-THC) is the principal active constituent (first isolated in 1964 by Gaoni and Mechoulam[1]); however, this term also now encompasses all those substances capable of activating cannabinoid receptors.

In animals, cannabinoid agonists produce a characteristic combination of four symptoms: hypothermia, analgesia, hypoactivity, and catalepsy[2]. In humans, where the majority of information relates to ingestion of the marijuana plant, the most familiar psychoactive effect is a state of mild euphoria (or “high”); however, cannabis also produces a wide range of other effects including an enhancement of sensory perception, tachycardia, antinociception, difficulties in concentration, suppression of nausea, and an increased appetite, together with impairment of linear thinking, memory, and locomotor and psychomotor skills (for detailed reviews see[3,4,5,6]). Adverse effects also include depersonalization and panic attacks, with frequent use by young adolescents contributing to an amotivational syndrome[3]. In terms of dependence, withdrawal symptoms from cannabis in man are thought to be mild and short lived; indeed, compared to cocaine, alcohol, opiates, and nicotine, marijuana has little addictive power, although dependence is observed in a significant minority of regular cannabis users. Chronic, high doses of cannabis may cause a subtle impairment of cognitive performance that can persist after cannabis use is discontinued, and these may be related to neurotoxic effects[7].

## PHARMACOLOGY: RECEPTOR AND NONRECEPTOR-MEDIATED ACTIONS IN THE CNS

Two types of G-protein-linked cannabinoid receptors ($CB_1$ and $CB_2$) have been identified using molecular and pharmacological approaches[8], with $CB_1$ receptors expressed predominantly in neurons of the brain, spinal cord, and peripheral nervous system and $CB_2$ receptors present mainly on immune cells. A splice variant of the $CB_1$ receptor, $CB_{1A}$, has been identified in brain[9] and exhibits all the properties of $CB_1$, but to a slightly attenuated extent[10]; however, the functional significance of this splice variant is questionable as other groups have not found evidence for its expression. The $CB_2$ receptor has only 44% overall nucleotide sequence identity with the $CB_1$ receptor. Unlike the $CB_1$ receptors, there is no evidence for $CB_2$ receptor binding, protein, or mRNA in the brain[11] although there are some functional data pointing to the presence of $CB_2$ receptors in cultured cerebellar granule cells[12]. Advances in cannabinoid pharmacology have generated a number of selective agonists and antagonists for these receptor subtypes (reviewed in detail elsewhere)[8,13].

While the majority of the physiological actions of cannabinoids in the CNS are mediated by activation of $CB_1$ receptors, there are some notable exceptions. Recent studies with $CB_1$ receptor knockout mice have shown some residual CNS actions of cannabinoids on synaptic transmission[14] and point to the existence of a novel type of G-protein-coupled cannabinoid receptor[15]; but this result remains to be confirmed using molecular cloning approaches. The endogenous cannabinoid anandamide also has activity at vanilloid receptors (VR1)[16], blocks directly the background $K^+$ channel TASK-1[17], and may directly interact with NMDA

receptors[18]. Likewise, cannabidiol activates VR1 receptors but also inhibits the cellular uptake and hydrolysis of anandamide[19]; indeed, some of the nonreceptor-mediated effects of cannabinoid compounds may be secondary to effects on metabolism of the active compounds. This distinction could be an important issue when working with cannabis extracts. Cannabinoids are reported to interact with 5HT-3 receptor channels at relatively low concentrations[20], and anandamide may directly interact with nicotinic acetylcholine receptors[21]. Anandamide also inhibits binding to the muscarinic acetylcholine receptors via a cannabinoid receptor–independent mechanism[22]. The synthetic, nonpsychoactive cannabinoid-like compound HU-211 directly antagonizes NMDA receptors but does not bind to cannabinoid receptors[23]. There is also evidence for nonreceptor-mediated neuroprotective (antioxidant) and cardiovascular effects of cannabinoids[24,25,26]. Nonreceptor-mediated responses to relatively low (up to 1 $\mu M$) concentrations of cannabinoid drugs include effects on neuronal uptake of certain neurotransmitters, inhibition of membrane-bound enzymes, and the perturbation of membrane phospholipids[27].

## CELLULAR LOCALIZATION OF $CB_1$ RECEPTORS

$CB_1$ receptors display a unique CNS distribution and are present in the mammalian brain at higher levels than most known G-protein-coupled receptors (GPCRs). The patterns of distribution of $CB_1$ receptors have been studied mainly with radioligand binding and autoradiography and with immunohistochemical techniques. Tritiated cannabinoid receptor ligands that have been the most widely used in binding assays or for autoradiography are the $CB_1$-selective [$^3$H]SR141716A, and [$^3$H]CP55940, [$^3$H]WIN55212-2, and [$^3$H]HU-243. The latter three are agonists that bind equally well to both $CB_1$ and $CB_2$ receptors. Whole-brain cannabinoid receptor density rivals that of glutamate[28] and GABA[29]. In certain regions, cannabinoid receptor levels are similar to those for dopamine and greatly exceed those for neuropeptide receptors. Computer-assisted densitometric analyses carried out on film autoradiographs taken from dog, guinea pig, rat, monkey, and human brains[30] show the highest levels of binding associated with the globus pallidus and substantia nigra pars reticulata and the molecular layers of the cerebellum and the dentate gyrus of the hippocampus. Regions showing strong binding include other areas of the hippocampal formation, the cerebral cortex, and the striatum. The hypothalamus, basal ganglia, solitary tract nucleus, and spinal cord show moderate levels of radioligand binding, with slight differences between species[30].

Immunocytochemical studies indicate that $CB_1$ receptor distribution resembles that of radioligand binding sites for cannabinoids, with relatively high levels of expression in areas such as the olfactory system, hippocampal formation, neocortex, and cerebellum[31,32,33,34]. The heterogeneity of $CB_1$ receptor expression relates to areas associated with the characteristic effects of cannabinoid agonists on motor coordination and posture, hypothermia, learning and memory, appetite, nausea, and antinociception, supporting the hypothesis that cannabinoid effects in the brain are mediated via $CB_1$ receptors. At the cellular level, $CB_1$ receptor immunoreactivity is particularly associated with nerve fiber systems and axon terminals, but with little neuronal somatic expression, which is compatible with a presynaptic site of action. In the rat hippocampus, an area that has been studied in much detail, axonal $CB_1$ receptor immunoreactivity has been associated mainly with presynaptic GABAergic nerve endings of cholecystokinin-containing basket cells[35,36,37], and there is limited[32] but controversial[31,35,38] immunohistochemical evidence for the expression of $CB_1$ receptors on glutamatergic cells; however, *in situ* hybridization studies suggest that $CB_1$ receptors may be present, but at much lower levels than on GABAergic neurons[39]. Antibodies raised against extracellular epitopes of $CB_1$ receptors can be used to study their surface expression in living cells (Fig. 1). Cell surface $CB_1$ receptor immunolabeling in hippocampal neurons is also expressed at high levels at GABAergic synaptic terminals, with no detectable labeling of glutamatergic neurons[40].

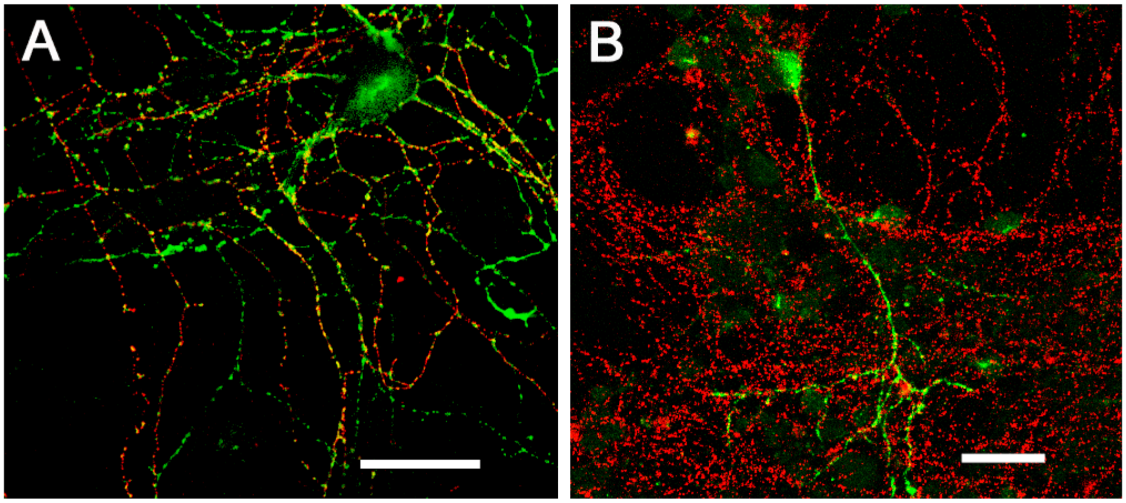

**FIGURE 1**. A comparison of cell surface $CB_1$ receptor immunoreactivity in cultured hippocampal neurons and cerebellar granule cells. (A,B) dual labeling of surface $CB_1$ receptors (red) and glutamate decarboxylase (GAD), a marker for inhibitory terminals/neurons (green). The extent of colocalization is shown by the yellow regions of overlap. Both cultures express strong, punctate $CB_1$ receptor immunoreactivity on fine fibers. Note the marked correspondence between $CB_1$ receptor label and clusters of GAD immunoreactivity in the cultured hippocampal neurons (A), but not in the cerebellar granule cells (B). An antibody raised against the N-terminus of the cloned rat $CB_1$ receptor was used to detect cell surface immunoreactivity. Scale bars, 20 μm.

## CANNABINOID SIGNAL TRANSDUCTION

Two of the most widely described intracellular effects of activation of $CB_1$ receptors in neurons are the inhibition of agonist-induced cyclic AMP (cAMP) formation[41] and blockade of inward $Ca^{2+}$ currents mediated by N- and P/Q-type $Ca^{2+}$ channels[42]. These characteristics are shared by a number of other G-protein-linked receptors (mGlu, muscarinic, and opioid) that modulate synaptic transmission. Moreover, these $Ca^{2+}$ channels are chiefly located presynaptically and are required for evoked neurotransmitter release. Other electrophysiological effects of cannabinoids include activation of A-type, inwardly rectifying[43], and inhibition of M-type[44] $K^+$ channels. Some of these effects on $K^+$ channels may be secondary to the formation of cAMP[41,43]. The actions of cannabinoids on ion channels are usually blocked by pretreatment of cells with pertussis toxin, suggesting an involvement of $G_{i/o}$; however, in cultured striatal neurons, pertussis toxin treatment unmasks a cannabinoid receptor–mediated stimulatory effect on cAMP accumulation, suggesting the activation of a novel signaling pathway involving a $G_s$-type G-protein[45]. Intracellular signaling pathways activated by cannabinoids in the CNS include the MAP kinase[46] and possibly c-Jun N-terminal kinase via a phosphoinositide 3'-kinase-dependent process[47]. Additionally, Netzeband et al.[48] have shown that $CB_1$ receptors can interact with phospholipase C in cerebellar granule cells, leading to the mobilization of intracellular $Ca^{2+}$.

The $CB_1$ receptor, like many other GPCRs, undergoes agonist-induced desensitization[49,50,51,52]. This process involves G-protein uncoupling in response to phosphorylation by G-protein-coupled receptor kinases (GRKs) and receptor internalization[51]. GRK-mediated receptor phosphorylation promotes the binding of beta-arrestins, which uncouple receptors from heterotrimeric G-proteins and target $CB_1$ receptors for internalization in clathrin-coated vesicles[50,51]. Studies of $CB_1$ receptor internalization have used transfected Chinese hamster ovary or AtT20 cells[49,50] and native receptors expressed on cultured hippocampal neurons or F11 cells[52]. Interestingly, the time course of internalization appears to be dependent on the cell type studied. The rate of internalization described for transfected cells or cell lines expressing $CB_1$ receptors is on the order of 10 to 30 min to achieve maximal levels, whereas it increases to between 5 and 16 h in hippocampal neurons maintained in primary culture[52]. This may reflect a difference in the internalization machinery between the cell types or between different subcellular compartments (axons vs. soma, for example).

Cell surface $CB_1$ receptor immunolabeling in hippocampal neurons is expressed at high levels at GABAergic synaptic terminals with no detectable labeling of somata[40]; however, GABAergic somata do express $CB_1$ receptor intracellular labeling in fixed and permeabilized cells, indicating that their site of synthesis is distant from their site of insertion into the plasma membrane. Likewise, in sensory neurons, cannabinoid $CB_1$ receptors are synthesized in somata and undergo axonal flow towards terminals, where they are inserted[53]. Following agonist exposure this situation is reversed with hippocampal $CB_1$ receptors being translocated back along axonal fibers towards the soma and proximal dendrites[52].

## CELLULAR PHYSIOLOGY

### Modulation of Synaptic Transmission

Cannabinoid actions can be classified into at least two types: (1) those mediated directly through cannabinoid receptors and (2) those mediated indirectly through other systems. Activation of cannabinoid $CB_1$ receptors modulates the release of a variety of neurotransmitters in the CNS (for a recent, detailed review, see[54]), including that of the principal excitatory and inhibitory neurotransmitters, glutamate and GABA, respectively, and also slower acting transmitters/neuromodulators such as the opioids[55,56], acetylcholine[57], dopamine[58], noradrenaline[59], and cholecystokinin[60]. The cellular mechanisms underlying these effects are thought to primarily reflect the actions of cannabinoids on presynaptic $Ca^{2+}$ or $K^+$ channels.

Cannabinoids modulate GABA release in a variety of CNS regions, including the hippocampus, basal ganglia, cerebellum, and brainstem. Studies have also shown inhibitory effects of $\Delta^9$-THC on GABA uptake in the hippocampus and basal ganglia[61,62]; however, electrophysiological investigations of GABAergic synaptic transmission have not confirmed this action. Anatomical invetigations have shown that high levels of $CB_1$ receptor immunoreactivity and mRNA are associated with GABAergic neurons[31,38,63]. In the hippocampus, neurochemical[38] and electrophysiological studies[37,64,65] have demonstrated that cannabinoids modulate GABA release from a subset of inhibitory neurons. In the basal ganglia (nucleus accumbens and striatum)[66,67,68,69,70], brainstem (rostral ventromedial medulla and periaqueductal gray)[71,72], and cerebellum[73,74], cannabinoids inhibit GABA release by a presynaptic mechanism. The majority of findings suggests that the actions of cannabinoids on GABA release are mediated via inhibition of presynaptic $Ca^{2+}$ channels (N or N/PQ)[54,65], although in some cases there may be a direct action on the release process itself[40,54,72].

Glutamatergic synaptic transmission is also modulated by cannabinoids in a number of key pathways. $CB_1$ receptor labeling has been detected on excitatory terminals in cultured cerebellar granule cells[75]. There is also good electrophysiological evidence that cannabinoid receptor activation inhibits glutamate release at the Purkinje cell–parallel fiber synapse in cerebellar slices[73,76,77,78]. In the striatum, glutamate release from cortical afferents is inhibited by $CB_1$ receptor activation[79]. Furthermore, in the midbrain periaqueductal gray, glutamatergic synaptic transmission is inhibited by cannabinoids[71]. In the nucleus accumbens, cannabinoids may modulate glutamate release from glutamatergic cortical afferents via a mechanism involving modulation of presynaptic $K^+$ channels[80] (but see[69]). Controversy exists with regard to excitatory synaptic transmission in the hippocampus with some studies showing an inhibition by cannabinoids[81,82] but others suggesting no effect[62,83]. These differences may reflect a developmental change in the sensitivity of glutamatergic synaptic transmission to cannabinoids[84] and/or species-dependent actions; thus, cannabinoids are without effect on glutamatergic synaptic transmission in adult rat hippocampal slices[83], but inhibit this activity in adult mouse hippocampus[82], neonatal tissue from rat[84] or mouse[82], and in cultured rat hippocampal neurons[81]. There is also no consensus regarding the expression of $CB_1$ immunoreactivity on pyramidal neurons[31,32]. Of interest is the recent suggestion that in the brain there is a new type of cannabinoid receptor that modulates glutamatergic synaptic transmission[14]. It remains to be demonstrated whether this can account for the observed discrepancies between $CB_1$ receptor expression and function.

There is a growing body of evidence suggesting that cannabinoids can modulate postsynaptic responses to glutamate and glutamatergic agonists. In the nucleus accumbens medium spiny neurons, evoked glutamatergic excitatory postsynaptic currents are inhibited by a postsynaptic mechanism[69]. Activation of AMPA receptors is modulated by cannabinoids in a *Xenopus* oocyte expression system[85]. Studies by Hampson et al.[18] have shown that the endogenous cannabinoid ligand anandamide has a dual action on NMDA receptor–mediated responses in a variety of CNS regions. It directly potentiates electrophysiological responses to NMDA, but inhibits NMDA receptor–mediated $Ca^{2+}$ elevations through a mechanism involving inhibition of voltage-gated $Ca^{2+}$ channels. Recent work in cultured cerebellar granule suggests that cannabinoids can enhance NMDA receptor–mediated $Ca^{2+}$ elevations via an indirect mechanism[48].

### Synaptic Plasticity

Cannabinoids inhibit various forms of synaptic plasticity in the hippocampus, including long-term potentiation (LTP)[82,86,87] and long-term depression (LTD)[82] via a $CB_1$ receptor–dependent mechanism. Within the cortex[88] and striatum[89], synaptic plasticity is also compromised by cannabinoids. The mechanism underlying these effects remains controversial. Misner and Sullivan[82] have suggested that cannabinoid modulation of hippocampal synaptic plasticity is mediated by the inhibition of glutamate release and that this can be overcome under conditions where the synaptic activation of NMDA receptors is enhanced ($Mg^{2+}$-free medium or postsynaptic membrane depolarization during the LTP- or LTD-induction protocol); however, Paton et al.[83] suggest that in some cases, LTP is blocked where glutamatergic synaptic transmission is unaffected. The relationship between synaptic plasticity and the effects of cannabinoids on inhibitory synaptic transmission are likely to be subtle as they influence only a subpopulation of interneurons. This might explain why cannabinoids do not in fact enhance synaptic plasticity, as would be expected with a more general reduction in inhibitory drive. The actions of cannabinoids on inhibitory synaptic transmission could be important in controlling the power of network oscillations, which are thought to be critical for learning and memory[37]. Studies with $CB_1$ receptor knockout mice suggest that endocannabinoids exert a tonic inhibitory effect on synaptic plasticity and that anandamide could be an endogenous messenger involved in the modulation of memory processes[90,91].

## ENDOCANNABINOIDS

The discovery of endogenous cannabinoid receptor ligands — anandamide (N-arachidonylethanolamine) and 2-arachidonylglycerol (2-AG) and its ether form, noladin ether — in the brain suggested the existence of a central endocannabinoid neuromodulatory system. Together with cannabinoid receptors, this cannabimimetic system is thought to have a widespread role in fine-tuning a variety of brain functions, including nociception, control of movement, memory, and neuroendocrine regulation. Recently it has also been suggested that this system is involved in brain development[92]. It is noteworthy that the highest levels of anandamide are expressed in the hippocampus[93].

Anandamide is released from neurons upon depolarization through a mechanism that requires $Ca^{2+}$-dependent cleavage from a phospholipid precursor in neuronal membranes[94]. The release of anandamide is followed by rapid uptake via a transporter into the cytosol and hydrolysis by the enzyme that inactivates endocannabinoids, fatty acid amide hydrolase (FAAH); indeed, the presence of $CB_1$ receptors/anandamide/FAAH in the thalamus, hippocampus, and cortex, or in the striatum, substantia nigra, and cerebellum, respectively, supports a role for the endogenous cannabinoid system in cognitive and motor responses. Studies with $CB_1$ receptor and FAAH knockout mice have also highlighted the importance of the endocannabinoid system in the control of normal CNS function, including retrograde synaptic inhibition in the hippocampus, long-term potentiation and memory, the development of opiate dependence, the control of appetite and pain perception[95,96].

Detailed neuroanatomical and electrophysiological analyses of mammalian nervous systems suggests that the $CB_1$ receptors are targeted to the presynaptic terminals of neurons; however, FAAH is preferentially located within the somatodendritic compartment of neurons that are postsynaptic to $CB_1$-expressing axon terminals. This localization is consistent with a model of cannabinoid signaling in which anandamide is synthesized by postsynaptic cells and acts as a retrograde messenger molecule to modulate neurotransmitter release from presynaptic terminals[97] (Fig. 2). However, some of the actions of cannabinoids on synaptic transmission may also be postsynaptic[18,98]. Recent experimental work suggests that endogenous cannabinoids mediate signals retrogradely from depolarized postsynaptic neurons to presynaptic terminals to suppress subsequent neurotransmitter release, driving the synapse into an altered state. In hippocampal neurons[94], depolarization of postsynaptic neurons and resultant elevation of $Ca^{2+}$ lead to a transient depolarization-induced suppression of inhibition (DSI). In cerebellar Purkinje cells, depolarization causes a transient suppression of both excitatory[78] and inhibitory[74] transmitter release from presynaptic terminals. The former is termed depolarization-induced suppression of excitation (DSE). Both DSI and DSE appear to share the same properties and may be a general and important mechanism by which the postsynaptic neuronal activity can influence the amount of transmitter release. Since it has been suggested that endocannabinoids released from a cell act over a short distance (~20 μm)[94], only cells in close proximity will be influenced.

## BEHAVIORAL EFFECTS AND THERAPEUTIC POTENTIAL

### Attention and Perception

Anecdotal and experimental evidence in humans suggests that marijuana intoxication produces only minor distortions in sensory awareness, including some reports of heightened sensory perception[3]. In monkeys, acute or chronic exposure to marijuana smoke had no serious deleterious effects on simple visual discrimination tasks[99]; however, there are reports of significant effects of cannabinoids on attention processes in both humans and animals. $\Delta^9$-THC produced dose-dependent effects on both the accuracy and latency of the responses to differential tone discrimination[100,101] and on signal detection performance in rats[102]. The performance of monkeys, trained to respond in a choice reaction time task, was significantly disrupted by acute exposure to marijuana smoke[103]. In humans, marijuana intoxication produced detrimental effects on both attention span and divided-attention assignments[104]. These data suggest that cannabinoid receptor activation does not appear to affect the performance of tasks that do not require focused attention or persistent detailed perception. On the other hand, these same

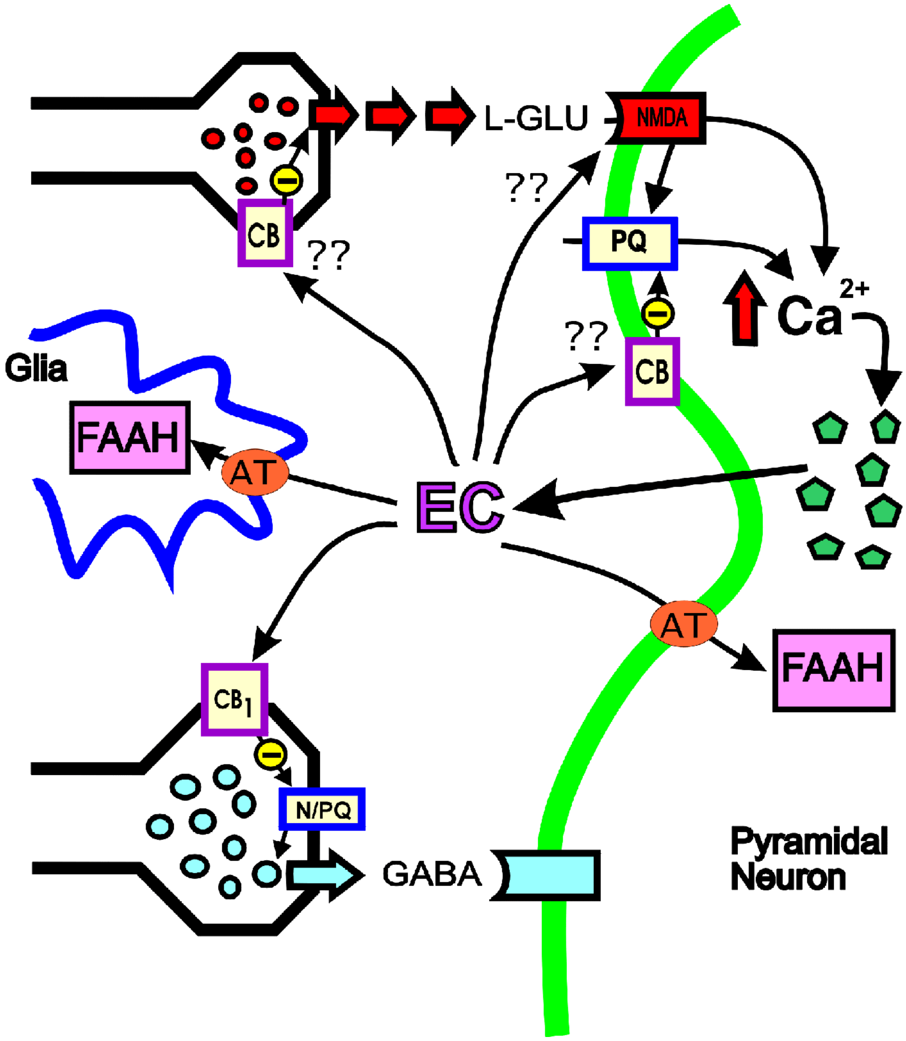

**FIGURE 2**. A model for the actions of endocannabinoids in the hippocampus. Strong excitation of pyramidal neurons via the release of L-glutamate (L-Glu) is very effective at increasing intracellular $Ca^{2+}$ levels, involving both depolarization (N/PQ voltage-gated $Ca^{2+}$ channels) and activation of NMDA receptors. It is suggested that this stimulates the release of endocannabinoids (EC), which act in a retrograde manner on both inhibitory (via $CB_1$ receptors) and excitatory terminals (possibly involving a novel cannabinoid receptor, CB). The postsynaptic activation of voltage-gated $Ca^{2+}$ channels (PQ) and NMDA receptors may also be modulated. Endocannabinoids are removed by a transporter (anandamide transporter, AT), possibly into both neurons and glia, where they are metabolized by fatty acid amide hydrolase (FAAH). The net outcome will be a balance between these excitatory and inhibitory actions, with effects on excitatory transmission necessary to prevent the system from becoming unstable.

discriminatory processes may become susceptible to the influence of cannabinoid agonists when more sustained or divided attention is necessary. In general, in animal models the outcome of cannabinoid receptor activation on attention or perception tasks is thought to resemble that of hippocampal lesions.

## Memory and Cognition

The main effects of cannabinoids on cognition in humans relate to the disruption of short-term memory[104,105], particularly with short-term amnesia and distortion of recently acquired information. These effects can be quite severe and dissociated from other effects of cannabinoids on cognition[106]. Experiments in animals also demonstrate cannabinoid-mediated memory deficits, and these are related to impairment of the function of the hippocampus, a structure that is intimately involved in the processes that underlie learning and memory[107]. Studies have shown that activation of cannabinoid receptors produces memory deficits similar to those produced by neurochemical lesions of the hippocampus[108]. Such lesions impair performance in short-term spatial memory tasks learned prior to the lesion. In rats, $\Delta^9$-THC reduced exploratory parameters and motor activity and caused more errors in maze tests and problems with information retention. This was associated with a reduction in noradrenaline levels in the hippocampus and increased dopamine levels in the amygdala[109]; however, this situation is unclear since low doses of anandamide (1 μg/kg) were found to improve impaired maze performance and increase hippocampal dopamine and noradrenaline levels in food restricted mice[110]. In the zebra finch, a discrete neural network within the telencephalon controls song learning, and this area shows dense radioligand binding with the cannabinoid agonist [$^3$H]-CP55940. The expression of $CB_1$ mRNA (5.5 kb) within the caudal telencephalon is altered during the course of vocal development and was shown by *in situ* hybridization to be expressed at high levels in areas involved in song learning and production, suggesting a potential role for cannabinoid signaling in zebra finch vocal development[111].

In conclusion, although there is little evidence of long-term memory disturbance, there is no definitive evidence that this, too, may not be affected. Some data suggest that there is no lasting or residual influence of cannabinoid exposure on memory processes and that the effects on short-term memory processes do not extend beyond the time that cannabinoids are present in the system[103,112]; nonetheless, cannabinoid neurotoxicity affecting the hippocampus may result in a more permanent impairment of cognitive performance[7].

## Locomotor Actions

Central cannabinoid receptors are densely located in the output nuclei of the basal ganglia (globus pallidus and substantia nigra pars reticulata), indicating a possible role in the regulation of extrapyramidal motor activity[113,114,115]. Furthermore, in the striatum cannabinoids modulate synaptic transmission and plasticity[79,89]. The cerebellum is also involved in the fine tuning of movement, and in this region cannabinoids inhibit excitatory and inhibitory synaptic transmission onto Purkinje neurons[73,74,76,77,78]; thus, locomotor-related activity is likely to be compromised by cannabinoids at a number of different levels in the CNS. It is suggested that the endogenous cannabinoid tone participates in the control of movement and, therefore, the central endocannabinoid system might play a role in the pathophysiology of movement disorders[116,117]; thus, cannabinoids might be useful in the treatment of tics in Tourette's syndrome, in the reduction of levodopa-induced dyskinesia in Parkinson's disease, or spasm associated with multiple sclerosis, and in the treatment of chorea in Huntington's disease. The extent to which the beneficial actions of cannabinoids in motor disorders are due to psychoactive or analgesic effects, however, remains to be established.

## Reward

Electrical or chemical stimulation of the medial forebrain bundle (MFB) can establish responses similar to those associated with natural rewards such as food or sexual contact, including intense self-stimulation in animals and euphoria in humans[118]. The reward/reinforcement circuitry of the mammalian brain consists of synaptically interconnected neurons associated with the MFB, linking the ventral tegmental area, nucleus accumbens, and ventral pallidum. Cannabinoids have been shown to increase the activity of dopaminergic neurons in the ventral tegmental area–mesolimbic pathway[118,119], circuits that are known to play a pivotal role in the neural substrates of drug addiction, withdrawal, and craving; however, these actions appear to be indirect as they do not to directly affect dopamine release itself[54]. In the nucleus accumbens, medium spiny GABAergic projection neurons receive synaptic input from both intrinsic GABAergic interneurons and extrinsic glutamatergic sources[69]. Both GABAergic and glutamatergic synaptic transmissions onto medium spiny neurons are inhibited by cannabinoids as well as other drugs of abuse[69,70], suggesting that this action may contribute to their rewarding properties.

## Appetite

Both marijuana and anandamide stimulate food intake and recent work is shedding light on the mechanism that underlies this effect. Endocannabinoids are implicated in the processes that regulate appetite in the hypothalamus[120], where they interact with leptin, a hormone critically involved in the central control of food intake and energy homeostasis. Following temporary food restriction, $CB_1$ receptor knockout mice eat less than their wild-type littermates, whereas the $CB_1$ antagonist SR141716A reduces food intake in wild-type, but not $CB_1$ knockout, mice[120]. The role of central cannabinoid systems in appetite regulation may also involve activation of reward systems and be mediated in part by opioidergic processes; thus, naloxone reduces the facilitory effect of $\Delta^9$-THC on feeding evoked by electrical stimulation of the lateral hypothalamus[121], and synergistic interactions between the effects of naloxone and SR141716A on nocturnal food (chow) intake tests have been documented[122]. From a therapeutic perspective, synthetic $\Delta^9$-THC (Marinol) has been used to stimulate the appetite of AIDS patients[4]. Cannabis may also be useful in the treatment of anorexia, but this action remains to be demonstrated in clinical trials. Clinical studies suggest SR141716A might be an effective antiobesity agent and has recently entered phase III clinical trials for this purpose (for more information see www.sanofi-synthelabous.com/excellence/).

## Pain

Analgesic effects of cannabis and cannabinoids have been demonstrated in most animal models of pain. These antinociceptive effects involve actions at a number of different levels, including peripheral sensory neurons, spinal cord, and central pathways[6,123]. In the brain and spinal cord a cannabinoid interaction with the opioidergic system may act to modulate the perception of painful stimuli[6]. Cannabinoids ameliorate pain by modulating rostral ventromedial medulla (RVM) neuronal activity in the brainstem in a manner similar to, but pharmacologically dissociable from, that of morphine[124]. The nucleus reticularis gigantocellularis pars alpha within the RVM is a major source of this descending modulation, and this system is activated in response to noxious stimulation[54,71]. Cannabinoids also inhibit synaptic transmission in the midbrain periaqueductal gray[72]. This area forms part of a descending antinociceptive pathway that, via the RVM, modulates nociceptive transmission at the level of the spinal cord[125]; thus, by acting at many different levels, cannabinoids may provide a useful alternative therapy for the treatment of pain, in particular in inflammatory and neuropathic pain[123], which do not respond well to conventional therapies.

## Neurotoxicity

An exciting new area of cannabinoid research is the potential of these compounds to modulate neurotoxicity. In the CNS, cannabinoids may protect neurons from toxic insults such as glutamatergic overstimulation, ischemia, and oxidative damage. Cannabinoid receptor activation is neuroprotective in several experimental models of cerebral hypoxia/ischemia and seizure activity[126,127]. A neuroprotective role for N-acylethanolamines, including anandamide, has also been suggested on the basis of their production at sites of neuronal damage or death[128]. In mice, the levels of endogenous 2-AG are also significantly elevated after closed head injury[129]; however, in neonatal rats, levels of 2-AG and other 2-monoacylglycerols are virtually unaffected following various models of *in vivo* neurodegeneration[128]. As a note of caution, relatively short periods of exposure to some cannabinoids, including $\Delta^9$-THC, are neurotoxic in cultured neurons, with shrinkage of neurons and DNA fragmentation in hippocampal tissue, an effect which is $CB_1$ receptor-mediated[7,130]. Chronic pretreatment with the cannabinoid agonist WIN 55212-2 can induce observable subcellular morphological changes, particularly in dendritic arrangements in the rat hippocampus[131]. There are also a number of unresolved issues that further complicate the role of cannabinoids in neuroprotection. First, the action of cannabinoids on glutamate release is controversial (see above). Second, some synthetic, nonpsychotropic cannabinoids such as HU-211 have NMDA receptor antagonist properties[23]. Third, cannabinoids have a multitude of seemingly contradictory actions; for example, activation of $CB_1$ receptors inhibits NMDA-induced intracellular $Ca^{2+}$ rises in the cortex but enhances similar responses in the cerebellum[18,48]. Furthermore, anandamide enhances NMDA-receptor-mediated synaptic transmission in the hippocampus via a direct interaction with the NMDA receptor *per se*[18], and certain cannabinoids may possess intrinsic antioxidant activity, thereby limiting neuronal damage via a receptor-independent pathway[26]. Fourth, interactions between adenosine, a widely studied neuroprotective agent, and cannabinoids have also been described[132].

## Epilepsy

Some data support the possible use of cannabis or cannabinoids in the treatment of epilepsy, but they can exert both convulsant and anticonvulsant effects[133,134]. The hippocampus is an area of the CNS particularly prone to the generation of seizures and is the subject of much study in this area. In the hippocampal slice preparation, anandamide, 2-AG, and WIN 55,212-2 attenuate epileptiform activity induced by exposure to $Mg^{2+}$-free medium or $Mg^{2+}$-free medium with elevated $K^+$[135,136]. Low $Mg^{2+}$-induced aberrant synaptic activity in cultured hippocampal neurons is also blocked by cannabinoids[81]. These actions of cannabinoids are sensitive to SR141716A, suggesting an involvement of $CB_1$ receptors. $\Delta^9$-THC can exert both convulsant and anticonvulsant actions in a model of cortical focal epilepsy[134] and can lower the seizure threshold following withdrawal[137]. A $CB_1$ receptor-mediated reduction in excitatory drive in the hippocampus could provide a mechanism for the prevention of excessive excitability leading to seizures; however, an associated inhibition of GABAergic synaptic transmission in the hippocampus would oppose this effect and may underlie some of the proconvulsant actions observed with $\Delta^9$-THC.

The nonpsychotic cannabinoids cannabidiol and HU-211 also exhibit anticonvulsant activity[138,139]; however, these actions may be indirect via effects on anandamide metabolism and NMDA receptor function, respectively[23,139]. At least some of the anticonvulsant actions of cannabinoids thus may be independent of $CB_1$ receptors. In conclusion, the therapeutic potential of cannabis and cannabinoids as anticonvulsants requires clarification.

## CONCLUSION

The role of cannabinoid systems in normal brain function and disease states is becoming clearer, and much detail has now been uncovered regarding their mechanisms of action. Considerable advances have been made in our understanding of cannabinoid receptor signaling pathways, their modulation of synaptic transmission and plasticity, the cellular targets of cannabinoids in different CNS regions and, in particular, the role of the endocannabinoid system; however, there are critical areas of controversy, including those related to the actions of cannabinoids in neurotoxicity and epilepsy. The presence of a new type of brain cannabinoid receptor is indicated and, if realized, will lead to both a further complexity in the actions of cannabinoids and to an exciting new therapeutic target. If all of the behavioral effects attributed to cannabinoids are mediated by $CB_1$ receptors, it will be more difficult to design clinical compounds that lack the undesirable psychotropic/cognitive/motor side effects. Alternative strategies using drugs that interfere with the synthesis, degradation, or uptake of endocannabinoids could prove to be useful therapeutically and would also help in our understanding of the brain endocannabinoid system. Given the current likelihood that cannabis will be legalized for medicinal use in the U.K. and possibly other countries, it is vital that we continue to unravel how its active constituents affect the CNS, both acutely and with sustained use.

## ACKNOWLEDGEMENTS

The authors would like to thank the MRC and the Wellcome Trust for financial support.
